# Cell type‐selective pathways and clinical associations of lysophosphatidic acid biosynthesis and signaling in the ovarian cancer microenvironment

**DOI:** 10.1002/1878-0261.12396

**Published:** 2018-11-15

**Authors:** Silke Reinartz, Sonja Lieber, Jelena Pesek, Dominique T. Brandt, Alina Asafova, Florian Finkernagel, Bernard Watzer, Wolfgang Andreas Nockher, Andrea Nist, Thorsten Stiewe, Julia M. Jansen, Uwe Wagner, Anne Konzer, Johannes Graumann, Robert Grosse, Thomas Worzfeld, Sabine Müller‐Brüsselbach, Rolf Müller

**Affiliations:** ^1^ Clinic for Gynecology Gynecological Oncology and Gynecological Endocrinology Center for Tumor Biology and Immunology (ZTI) Marburg Germany; ^2^ Center for Tumor Biology and Immunology (ZTI) Institute of Molecular Biology and Tumor Research (IMT) Marburg Germany; ^3^ Metabolomics Core Facility Philipps University Marburg Germany; ^4^ Institute of Pharmacology Marburg Germany; ^5^ Genomics Core Facility Philipps University Marburg Germany; ^6^ Clinic for Gynecology Gynecological Oncology and Gynecological Endocrinology UKGM Marburg Germany; ^7^ Biomolecular Mass Spectrometry Max‐Planck‐Institute for Heart and Lung Research Bad Nauheim Germany; ^8^ German Centre for Cardiovascular Research (DZHK) Kerckhoff Klinik Bad Nauheim Germany; ^9^ Department of Pharmacology Max‐Planck‐Institute for Heart and Lung Research Bad Nauheim Germany

**Keywords:** autotaxin, lipid signaling, lysophospholipid, macrophage, ovarian cancer, phospholipase

## Abstract

The peritoneal fluid of ovarian carcinoma patients promotes cancer cell invasion and metastatic spread with lysophosphatidic acid (LPA) as a potentially crucial mediator. However, the origin of LPA in ascites and the clinical relevance of individual LPA species have not been addressed. Here, we show that the levels of multiple acyl‐LPA species are strongly elevated in ascites versus plasma and are associated with short relapse‐free survival. Data derived from transcriptome and secretome analyses of primary ascite‐derived cells indicate that (a) the major route of LPA synthesis is the consecutive action of a secretory phospholipase A_2_ (PLA_2_) and autotaxin, (b) that the components of this pathway are coordinately upregulated in ascites, and (c) that CD163+CD206+ tumor‐associated macrophages play an essential role as main producers of PLA_2_G7 and autotaxin. The latter conclusion is consistent with mass spectrometry‐based metabolomic analyses of conditioned medium from ascites cells, which showed that tumor‐associated macrophages, but not tumor cells, are able to produce 20:4 acyl‐LPA in lipid‐free medium. Furthermore, our transcriptomic data revealed that LPA receptor (*LPAR*) genes are expressed in a clearly cell type‐selective manner: While tumor cells express predominantly *LPAR1‐3*, macrophages and T cells also express *LPAR5* and *LPAR6* at high levels, pointing to cell type‐selective LPA signaling pathways. RNA profiling identified cytokines linked to cell motility and migration as the most conspicuous class of LPA‐induced genes in macrophages, suggesting that LPA exerts protumorigenic properties at least in part via the tumor secretome.

**Abbreviations**
AA: arachidonic acid
FBS: fetal bovine serum
HGSC: high‐grade serous adenocarcinoma
LC‐MS/MS: liquid chromatography–tandem mass spectrometry
LOD: limit of detection
LPA: lysophosphatidic acid
LPC: lysophosphatidylcholine
MeOH: methanol
OC: ovarian cancer
PLA2: phospholipase A2
PMA: phorbol myristate acetate
qRT‐PCR: quantitative reverse transcriptase PCR
RFS: relapse‐free survival
RNA‐Seq: RNA sequencing
sPLA2: secretory phospholipase A2
TAM: tumor‐associated macrophage
TAT: tumor‐associated T cell

## 1 Introduction

Lysophosphatidic acid (LPA) is a lipid mediator with multiple functions in tumor growth and progression, including ovarian carcinoma (OC) (Chun *et al*., 2013a; Willier *et al*., 2013). Its most frequent and aggressive form is high‐grade serous ovarian carcinoma (HGSC), which accounts for approximately 75% of all ovarian malignancies (Committee on the State of the Science in Ovarian Cancer Research, 2016). HGSC is usually detected at an advanced stage characterized by widespread peritoneal metastases, which is the main reason for its dire prognosis.

A hallmark of OC is its tumor microenvironment, which is unique among all cancer entities (Worzfeld *et al*., 2017). It is composed of anatomically and functionally different compartments, that is, the solid tumor masses with the invaded surrounding host tissues (most notably the omentum) and the peritoneal fluid, which frequently occurs as ascites at advanced stages. The malignancy‐associated peritoneal fluid contains large numbers of tumor and immune cells, which interact to produce, and respond to, a plethora of mediators with metastasis‐promoting and immune‐suppressive properties. Among these, LPA plays a prominent role (Westermann *et al*., 1998; Xu *et al*., 1995), although potential associations between ascites levels of LPA, OC progression, and clinical outcome have not been analyzed to date.

Lysophosphatidic acid has been reported to enhance the adhesion, migration, invasion, and metastatic spread of OC cells (Bian *et al*., 2004; Kim *et al*., 2006; Li *et al*., 2009; Ren *et al*., 2006; So *et al*., 2004; Symowicz *et al*., 2005), to stimulate their epithelial‐to‐mesenchymal transition (Burkhalter *et al*., 2015; Ha *et al*., 2016), to promote sphere formation, expression of stemness‐associated genes, and tumor‐initiating properties (Seo *et al*., 2016) and to enhance OC cell survival and resistance to anticancer drugs (Seo *et al*., 2016; Tanyi *et al*., 2003a,b; Vidot *et al*., 2010). LPA also acts on OC‐associated host cells, for example, by inducing the differentiation of mesenchymal stem cells to cancer‐associated fibroblasts (Jeon *et al*., 2010). A number of LPA‐induced target genes with functions in tumor progression have been identified, including the genes for Cox‐2 (Symowicz *et al*., 2005), IL‐6 (Fang *et al*., 2004), IL‐8 (Fang *et al*., 2004; Schwartz *et al*., 2001), and Gro‐α (Lee *et al*., 2006).

Lysophosphatidic acid does not represent a single molecular entity, but a class of lipids composed of a glycerol backbone with a saturated or unsaturated fatty acid in the sn1 or sn2 position and substituted with a phosphate group in sn3 (Mills and Moolenaar, 2003). LPA species typically found in plasma or ascites harbor 16:0, 18:0, 18:1, 18:2, and 20:4 fatty acids linked via ester bonds in acyl‐LPAs or via ether bonds in alkyl‐LPAs. Alkyl‐LPAs are also present in blood as constituents of the plasmalogen fraction (Wallner and Schmitz, 2011) and in ascites (Lu *et al*., 2002; Xu *et al*., 2004) and, like acyl‐LPA, have been reported to exert tumor‐promoting activities.

Extracellular LPA is generated from phospholipids by the consecutive action of two enzymes via two pathways, that is, (a) a secretory phospholipase A_1_ or A_2_ (sPLA_1_ or sPLA_2_) followed by the lysophospholipase D autotaxin or (b) a phospholipase type D followed by an sPLA_1_ or sPLA_2_ (Chun *et al*., 2013b; Houben and Moolenaar, 2011). Autotaxin has been suggested to play a pivotal role in OC pathogenesis (Gaetano *et al*., 2009; Seo *et al*., 2016) and chemoresistance (Vidot *et al*., 2010).

Lysophosphatidic acid signals through six G protein‐coupled receptors, termed LPAR1‐6, which trigger both overlapping and distinct signaling pathways, that is, signaling via Gα_12/13_ to RAC/RHO, via Gα_i/o_ to phospholipase C, phosphatidylinositol 3‐kinase and RAS, via Gα_q/11_ to PLC and via Gα_s_ to adenylate cyclase (Yung *et al*., 2014). The classical LPA receptors, LPAR1‐3, belong to the endothelial cell differentiation gene (EDG) subfamily of GPCRs. The other three LPA receptors, that is, LPAR4 (P2Y9), LPAR5 (GPR92), and LPAR6 (P2Y5), are structurally more closely related to the purinergic receptors rather than the EDG family. Intriguingly, different LPAR subtypes may have opposing functions in tumor cells, as described, for instance, for the role of LPAR1 and LPAR2 in the LPA‐triggered migration of pancreatic cancer cells (Komachi *et al*., 2009). LPA also directly activates the TRPV1 ion channel through a C‐terminal binding site, which is specific for the 18:1 species (Nieto‐Posadas *et al*., 2011).

The goal of the present study was to gain detailed insight into the clinical relevance of individual LPA species and cell‐selective pathways of LPA signaling in OC. In view of its clinical prevalence and poor prognosis, we focused our research on HGSC.

## 2 Materials and methods

### 2.1 Patients

Ascites and peripheral blood were collected from patients with HGSC or benign conditions prior to surgery at Marburg University Hospital (Table S1). Cell‐free ascites and plasma were cryopreserved at −80 °C. The collection and the analysis of human materials were approved by the ethics committee at Philipps University (reference number 205/10). Donors provided written consent in accordance with the Declaration of Helsinki. Tumor cell spheroids were visualized by phase‐contrast microscopy, and numbers were categorized as in Table S1. Single tumor cells, TAM and TAT were quantified by flow cytometry using antibodies specific for EPCAM (tumor cells), CD45 (immune cells), CD14 (TAMs), and CD3 (TATs) as described (Reinartz *et al*., 2016).

### 2.2 Cell culture

Tumor cell spheroids, tumor‐associated macrophages (TAMs), and tumor‐associated T cells (TATs) were isolated from HGSC ascites as previously reported (Reinartz *et al*., 2016; Worzfeld *et al*., 2018). Tumor cell cultures were established from spheroids in ascites according to a recently described cell culture system for culturing primary cells from patient‐derived spheroids (OCMI‐37, OCMI‐38, and OCMI‐91 cells), which allows for the propagation of HGSC cells over long periods of time in the absence of a culture‐induced crisis or genetic alterations compared to the original tumor (Ince *et al*., 2015). In brief, patient‐derived spheroids were cultured on a mixed‐charged surface (Primaria culture dishes, Corning) in a special medium (OCMI) consisting of equal volumes of DMEM/Ham's F12 and M199 medium (Millipore, Darmstadt, Germany) supplemented with 2 mm glutamine, 20 μg·mL^−1^ insulin, 10 mm HEPES (pH 7.4), 10 μg·mL^−1^ transferrin, 0.2 pg·mL^−1^ triiodothyronine, 5 μg·mL^−1^ o‐phosphoryl ethanolamine, 8 ng·mL^−1^ selenous acid, 25 ng·mL^−1^ all‐trans retinoic acid, 500 ng·mL^−1^ hydrocortisone, 25 ng·mL^−1^ cholera toxin (all from Sigma‐Aldrich, Taufkirchen, Germany), 10 ng·mL^−1^ epidermal growth factor (Gibco, Carlsbad, CA, USA), 5 μg·mL^−1^ linoleic acid (Cayman Chemicals/Biomol, Hamburg, Germany), and 5% FBS (Gibco). The HGSC cell line OVCAR‐8 (Hernandez *et al*., 2016; Schilder *et al*., 1990) was obtained from the NIGMS Human Genetic Cell Repository of the NIH and cultured in RPMI 1640 (Life Technologies, Carlsbad, CA, USA) complemented with 10% FBS (Sigma). THP‐1 monocytic cells (Tsuchiya *et al*., 1980) were purchased from LGC Germany (ATCC, TIB202) and cultured in RPMI 1640 supplemented with 10% heat‐inactivated FBS and 0.05 mm 2‐mercaptoethanol (Gibco). THP‐1 cells were differentiated to macrophages by adding phorbol myristate acetate (PMA) for 4 h followed by 5 days of normal medium prior to harvesting.

### 2.3 Conditioned medium from tumor cells and TAMs

To examine the role of TAMs and tumor cells in generating extracellular LPA, patient‐derived TAMs and OCMI tumor cells were cultured in OCMI medium (without FBS) supplemented with 50% ascites (pool of five patients) for 24 h, 37 °C, 5% CO_2_. After starvation in ascite‐free OCMI medium containing 0.1% fatty acid‐free BSA for an additional 24 h, 20 μm of exogenous LPC (equimolar mixture of 18:1 and 16:0) or solvent (control) was added and harvested at different time points (as indicated). Samples were stored at −80 °C for LC‐MS/MS analysis of LPA and LPC. Lipids used for cell culture experiments (16:0‐LPC, 18:1‐LPC) were purchased from Avanti Polar Lipids (Alabaster, AL, USA).

### 2.4 Three‐dimensional Matrigel invasion

Transwell inserts (Thincerts, Greiner Bio‐One; 24‐well, 8 μm pore size) were coated with 50 μL growth factor reduced Matrigel (Corning) at 5 μg·μL^−1^, and cell invasion was analyzed essentially as described (39). Briefly, 15 000 tumor cells were seeded on transwell inserts and allowed to adhere for 1 h. Thincerts were inverted, and serum‐free medium containing individual LPA species (or 5% FBS as positive control) was added to the top compartment. Serum‐free medium was added to the lower compartment. Cells were fixed with 8% formaldehyde after 24 h, followed by staining of F‐actin with Alexa 555‐labeled phalloidin and DNA with Sytox green. Cell invasion was analyzed with a confocal microscope (Zeiss LSM 700; Zeiss, Jena, Germany). A tile scan with nine sections located around the center of the thincert was done followed by a quantification of invaded (cells at a distance of approximately 20 μm from the transwell membrane) versus noninvaded cells.

### 2.5 Two‐dimensional migration assay

Chemotactic migration was quantified using a Boyden chamber transwell assay (Yang *et al*., 2005). The assay was performed with OC cells in the presence of different LPA species in serum‐free OCMI medium (or 5% FBS as positive control) in the lower chamber as chemoattractants. Cells were seeded on filters (uncoated, 8.0 μm pore size; BD Biosciences) in 24‐well companion plates (BD BioSciences, Franklin Lake, NJ, USA) at 50 000 cells per filter in 300 μL medium. The companion plate was equilibrated at 37 °C in a 5% CO_2_ incubator for 30 min prior to the addition of chemoattractants for 20 h. Filters were stained with Crystal Violet solution (Sigma‐Aldrich; 1:10) for 10 min and evaluated under a Leica DMI3000B microscope (Leica, Wetzlar, Germany) at 5× magnification.

### 2.6 Chemicals for lipid LC‐MS/MS

16:0‐LPA, 18:0‐LPA, 16:0‐alkyl‐LPA, 18:0‐alkyl‐LPA, 18:1‐alkyl‐LPA, 16:0‐LPCs, 18:1‐LPC as well as 17:0‐LPA and LPC 17:0 as internal standards were purchased from Avanti Polar Lipids. LPAs 18:2, 18:3, 20:0, 20:4 were obtained from Echelon Biosciences, Inc. (Salt Lake City, UT, USA) and LPA 18:1 from Cayman Chemical Company (Ann Arbor, MI, USA). LCMS grade water, formic acid (HCOOH), chloroform, and ammonium formate were purchased from VWR International or Merck (both Darmstadt, Germany) and LCMS grade methanol (MeOH) was purchased from Honeywell (Seelze, Germany).

### 2.7 Preparation of standards for lipid LC‐MS/MS

17:0‐LPA was used as internal standard for LPA measurements. Standard LPA solutions (16:0, 18:0, 18:1, 18:2, 18:3, 20:4, alkyl‐16:0, alkyl‐18:0, and alkyl‐18:1) were made in MeOH. To obtain LPA standard curves, 10 μL of LPA standards in different concentrations (0–30 μm) was mixed with 10 μL of 17:0‐LPA solution (internal standard, 10 μm). To prevent matrix effects and reducing carryover of standard components, 100 μL ascites (with very low LPA concentration) was also added to each calibration sample. Proteins were precipitated by adding 1.5 mL methanol.

For LPC measurements, 17:0‐LPC was used as internal standard. For LPC standard curves, 5 μL of LPC standards (16:0, 18:0 and 18:1) in different concentrations (0–60 μm) were mixed with 990 μL of 17:0 LPC (internal standard, 0.01 μm) in methanol. To prevent matrix effects and reducing carryover of standard components, 5 μL ascites (with very low LPC concentration) was also added to each calibration sample. Further calibration sample preparation was done just as described for the samples. ESI‐MS was performed, and the intensity ratios (standard versus internal standard) were plotted against molar ratios (standard versus internal standard).

### 2.8 Sample preparation for lipid LC‐MS/MS

Quantitative determination of alkyl‐ and acyl‐LPA as well as acyl‐LPC was performed in ascites or plasma samples. All samples were centrifuged at 3300 g (Multifuge, Heraeus GmbH, Hanau, Germany) for 10 min. For LPA determination, ten microliters of 17:0 LPA (10 μm in MeOH) was added to 100 μL of the cell‐free centrifugates followed by 1.5 mL MeOH for protein precipitation. For LPC determination, on the other hand, 5 μL of the ascites or plasma centrifugates was mixed with 990 μL 17:0 LPC (0.01 μm in MeOH). All samples were vortexed for 2 min and centrifuged at 3300 g for 10 min. The precipitate‐free upper phase was transferred to a glass vial and dried under vacuum (RVC2‐25 CD plus, Christ GmbH, Osterode, Germany). The dried lipids were resuspended in 100 μL chromatography solvent B (1% HCOOH in MeOH with 5 mm ammonium formate) and vortexed thoroughly with assistance of sonication in an ultrasonic water bath for 2 min at room temperature. The samples were transferred into autosampler vials with inserts and centrifuged again for 10 min, prior to LC‐MS/MS analysis.

### 2.9 Instrumentation and analytical conditions for lipid LC‐MS/MS

ESI‐MS and tandem mass spectrometry (MS/MS) analyses were performed using a Sciex 5500 QTRAP mass spectrometer equipped with an ESI source. The samples were delivered into the ESI source using an Agilent 1290 system with LC pump, autosampler, and column oven. Injection volume was 5 μL. Chromatographic separations were performed using a Kintex C18 (2.1 × 50 mm, 2.5 μm; Phenomenex, Aschaffenburg, Germany) LC column at a temperature of 50 °C. The mobile phases used for all experiments were as follows: (a) MeOH:H_2_O:HCOOH (58:41:1 v/v) and (b) MeOH:HCOOH (99:1 v/v) both with 5 mm ammonium formate. The flow rate was set to 300 μL·min^−1^. The optimized elution gradient conditions were selected as follows: initial hold time with 100% A for 1 min, linear gradient from 0% to 100% B in 6 min, 6 min at 100% B, back to the starting conditions in 0.01 min and hold for 3 min. All quantitative MS analyses were performed in the multiple reaction monitoring (MRM) mode. LPAs were analyzed in the negative mode, whereas LPCs were analyzed in positive mode both with optimized MS/MS settings for each substance. The mass transitions and retention times used for quantification are summarized in Table S2. To prevent carryover, samples and blanks were measured alternately. Additionally, the injection needle was flushed thoroughly with methanol:formic acid (95:5).

### 2.10 Validation of LPA analysis by lipid LC‐MS/MS

The analytical LPA method was validated for selectivity, linearity, precision, and accuracy. The selectivity of the method was determined by comparing chromatograms of extracted blank samples to samples spiked with analytes to ensure that it was free of interferences at the retention times. The intraday precision and accuracy were determined within 1 day by analyzing five samples replicates at concentrations of 0.04, 0.4, 0.75, 1.1, and 1.5 μm for each compound. The accuracy of the assay was defined as the absolute value of the ratio of calculated mean values of the quality control samples to their respective nominal values, expressed as percentages. The interday precision and accuracy were determined on 5 separate days at identical concentrations. A signal‐to‐noise ratio of three is generally accepted for estimating the limit of detection (LOD) and a signal‐to‐noise ratio of ten is used for estimating limit of quantitation (LOQ). Based on residual standard deviation of the response and the slope, the LOD and LOQ of all compounds ranged between 0.04–1.4 nm and 0.2–4.6 nm, respectively. An eight‐point standard curve ranging from 0 to 30 μm of a mixture of all LPA lipids was used for determination of linearity, precision, and accuracy of the analytical method. A good linearity was obtained with r² always larger than 0.99. Intraday precision (CV) ranged from 0.9 to 12.8. Only the lowest concentration of some LPAs (16:0, 18:2 and 20:4) showed higher CVs. The intraday accuracy (RE) ranged from 104% to 125%, interday precision (CV) from 1.1 to 25.7, and interday accuracy (RE) from 105% to 128% (129% to 142% for some 16:0, 18:2, and 20:0 LPAs at very low concentrations).

### 2.11 qRT‐PCR

Isolation of RNA and qRT‐PCR were carried out as described (Reinartz *et al*., 2016; Rohnalter *et al*., 2015). *L27* was used for normalization. Results were evaluated by the Cy0 method (Guescini *et al*., 2008). The following primers were used:

RPL27_fw: 5′‐AAAGCTGTCATCGTGAAGAAC
RPL27_rv: 5′‐GCTGTCACTTTGCGGGGGTAG
AREG_fw: 5′‐TTTCAAAATTTCTGCATTCACG
AREG_rv: 5′‐ACTTTTCCCCACACCGTTC
BMP6_fw: 5′‐GTGAACCTGGTGGAGTACG
BMP6_rv: 5′‐CCTCACCCTCAGGAATCTG
OSM_fw: 5′‐GGACCCTATATACGTATCCAAGGC
OSM_rv: 5′‐GCATTGAGGGTCTGCAGG
THBS1_fw: 5′‐GTTGGCCCAGCGACTCTG
THBS1_rv: 5′‐GGTTGTTGAGGCTATCGCAG

### 2.12 Analysis of transcriptomes

RNA‐Seq of LPA‐treated cells was performed as described previously (Reinartz *et al*., 2016; Worzfeld *et al*., 2018). Data for tumor cells, TAMs, and TATs from HGSC ascites are from previous publications (Reinartz *et al*., 2016; Worzfeld *et al*., 2018). RNA‐Seq data were deposited at EBI ArrayExpress (accession numbers E‐MTAB‐7113).

### 2.13 Proteomic analysis of secretomes

Secretomes were determined by LC‐MS/MS of conditioned medium from short‐term cultures of primary cells isolated from HGSC ascites as described (Worzfeld *et al*., 2018). Data for tumor cells and TAMs were derived from our published datasets (Worzfeld *et al*., 2018). Data for TATs were obtained analogously. Proteomic data were deposited at PRIDE (accession numbers PXD008047).

### 2.14 Statistical analyses

Comparative data were statistically analyzed by unpaired Student's *t*‐test (two‐sided, equal variance) unless indicated otherwise. Cell type specificities (fold change) were analyzed by the bootstrapping method. Box plots depicting medians (line), upper and lower quartiles (box), range (whiskers) and outliers/fliers (diamonds) were constructed using the Seaborn boxplot function. Correlations were analyzed using the scipy.stat functions. Associations with relapse‐free survival (logrank test), hazard ratio (HR) and median survival times were analyzed using the Python Lifelines KaplanMeierFitter and CoxPHFitter functions. Data associating gene expression with overall survival (OS) were retrieved from the PRECOG database (https://precog.stanford.edu; Gentles *et al*., 2015), KM‐Plotter version 2017 (http://kmplot.com; Gyorffy *et al*., 2012) and The Cancer Genome Atlas (TCGA; Cancer Genome Atlas Research Network, 2011).

## 3 Results

### 3.1 Elevated levels of LPA species in ascites

**Figure 1:**
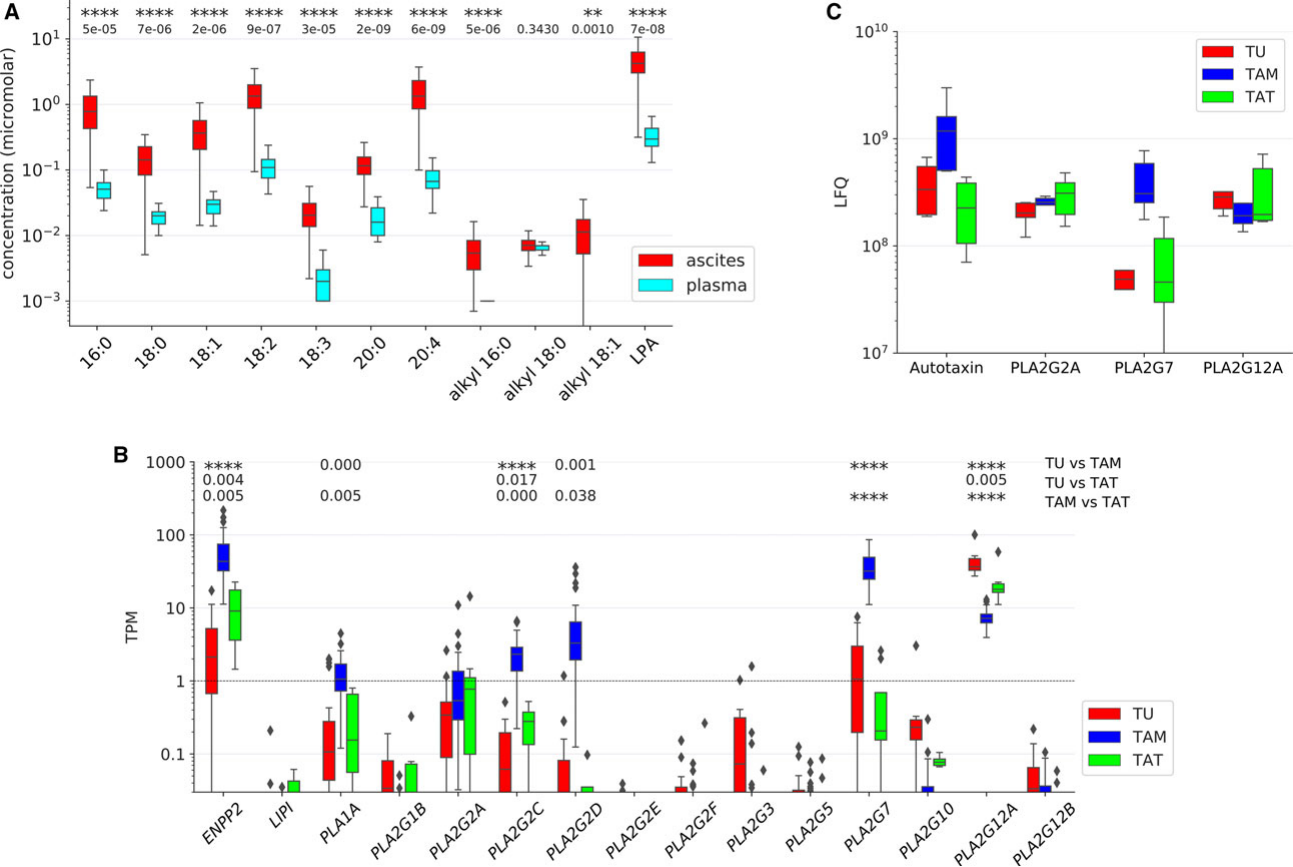
LPA and LPA‐generating enzymes in HGSC ascites. (A) Levels of LPA species in ascites and matched plasma samples from HGSC patients (*n* = 19). **: *P* < 0.01, ****: *P* < 0.0001 by paired *t*‐test. LPA: sum of all LPA species determined. (B) Expression of genes coding for LPA‐generating enzymes in tumor cells (TU) (*n* = 23), TAMs (*n* = 32), and TATs (*n* = 8) from HGSC ascites (RNA‐Seq data). (C) Secretion of LPA‐generating enzymes by tumor cells (TU), TAMs, and TATs from HGSC patients. Conditioned medium from primary cells cultured for 5 h in protein‐free medium was analyzed by LC‐MS/MS (*n* = 5 for each cell type). Boxplots show medians (horizontal line in boxes), upper and lower quartiles (box), and range (whiskers). *: *P* < 0.05, **: *P* < 0.01, ***: *P* < 0.001, ****: *P* < 0.0001 by unpaired *t*‐test.

We determined the levels of the seven most abundant acyl‐LPA species (16:0, 18:0, 18:1, 18:2, 18:3, 20:0, 20:4) and three alkyl‐LPAs (16:0, 18:0, 18:1) in 91 ascites samples from HGSC patients, 19 matched plasma samples, and 11 plasma samples from patients with nonmalignant diseases (ovarian cysts, myomatosis uteri) by LC‐MS/MS. While we found no statistically significant differences between the latter two cohorts (Fig. S1), the levels of all LPA species were higher in ascites (*n* = 91) compared to plasma (*n* = 30; Fig. S2). Comparison of 15 matched ascites and plasma samples also yielded highly significant differences (paired t‐test; Fig. 1A). Median total LPA levels (i.e., sum of all the levels for all LPA species analyzed) were 4.3 μm in ascites (maximum 22.8 μm) versus 0.36 μm in plasma (maximum 0.82 μm), corresponding to an ~12‐fold median enrichment). Differences between ascites and plasma were in the range of 5‐ to 28‐fold (medians) for individual acyl‐LPAs, and < 7‐fold or not determinable (due to low concentrations) for alkyl‐LPAs (Fig. 1A and Fig. S2). The highest ascite levels were found for 20:4 and 18:2 acyl‐LPAs (median 1.34 μm each), followed by 16:0 and 18:1 acyl‐LPAs (median 0.78 and 0.37 μm, respectively), with maximum concentrations of up to 8 μm in some patients (Fig. 1A). In contrast, all 3 tested alkyl‐LPA species were detectable only at low levels in ascites (< 0.1 μm). The levels of individual LPA species were strongly correlated (Spearman rho > 0.6–0.9; Fig. S3), except for 20:0 acyl‐LPA and 18:0 alkyl‐LPA, pointing to common metabolic pathways for most LPA species.

### 3.2 LPA‐generating enzymes in ascites

**Figure 2:**
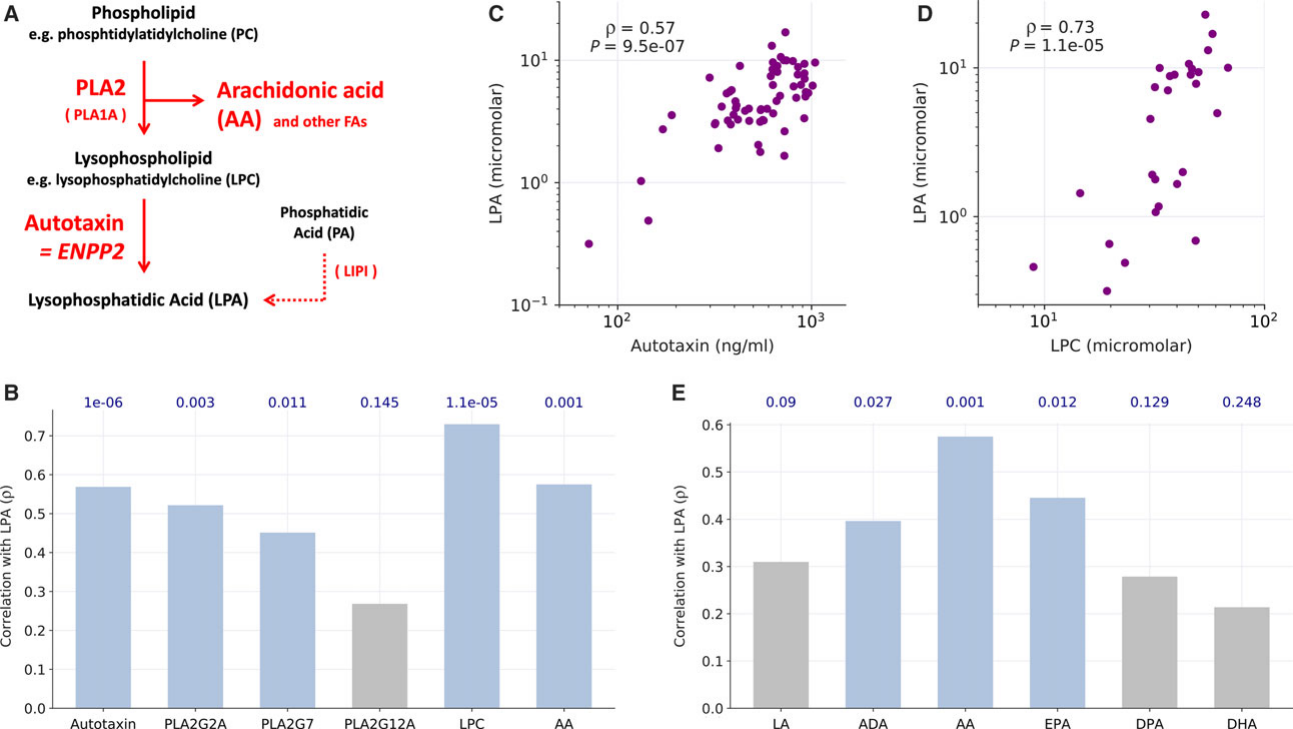
Correlation of metabolites and enzymes involved in the generation of LPA in HGSC ascites. (A) Schematic summary of LPA biosynthesis in HGSC ascites based on the data in Fig. 1. (B) Spearman correlation of the ascites levels of the indicated metabolites and enzymes. LPA: sum of all LPA species determined. (C, D) Dot plots illustrating the correlation of LPA concentration with the levels of autotaxin and LPC in ascites. (E) Spearman correlation of the levels of LPA and the most abundant PUFAs in ascites. ADA, docosatetraenoic acid (adrenic acid); DHA, docosahexaenoic acid; DPA, docosatetraenoic acid; EPA, eicosapentaenoic acid; LA, linoleic acid. Blue in panels B and E: significant (*P* ≤ 0.05), gray: not significant. Correlation *P*‐values are shown above the bars.

To identify the enzymes involved in the generation of LPA in HGSC ascites, we determined the level of RNA expression of all potentially involved type A1, A2, and D phospholipases (Quach *et al*., 2014). Toward this end, we analyzed RNA‐Seq data for the predominant cell types in ascites, that is, tumor cells, TAMs, and TATs. As shown in Fig. 1B, three enzyme‐encoding genes showed the highest expression, that is, *ENPP2* (autotaxin) in TAMs, *PLA2G7* in TAMs and *PLA2G12A* in all three cell types. In contrast, both genes coding for type A1 phospholipases *(LIPI, PLA1A)* were expressed at very low level, if at all, in any cell type. The TAM‐selective expression of autotaxin and PLA_2_G7 and the cell type‐independent high expression of PLA_2_G12A were confirmed analyzing the secretome of patient‐derived tumor cells, TAMs, and TATs in short‐term cultures (conditioned medium) by LC‐MS/MS‐based proteomics (Fig. 1C). However, in contrast to the RNA‐Seq data we also found high concentrations of PLA_2_G2A in the conditioned medium from all three cell types (Fig. 1C). It is possible that the RNA‐Seq data underestimate the expression of PLA_2_G2A, which may be due to a highly efficient translation of the *PLA2G2A* mRNA, a high stability of the PLA_2_G2A enzyme or a problem related to the RNA‐Seq methodology. Taken together, these observations lead to the conclusion that LPA in ascites is generated from phospholipids mainly by the consecutive action of a secretory PLA_2_ and autotaxin rather than the cleavage of phosphatidic acid by type A1 phospholipases (Fig. 2A). Our data also point to a prominent role for TAMs in this metabolic pathway as the main producers of autotaxin and PLA_2_G7.

Consistent with this conclusion, Spearman correlation analysis revealed a significant correlation of LPA with autotaxin, PLA_2_G2A, and PLA_2_G7 levels in ascites, with the best correlation observed for autotaxin (ρ = 0.58; Fig. 2B,C). We also measured the ascites levels of the PLA_2_‐generated phospholipid cleavage product and acyl‐LPA precursor lysophosphatidylcholine (LPC) by LC‐MS/MS and found a strong correlation with LPA levels (ρ = 0.72; Fig. 2B,D). The most common fatty acid in the sn2 position of phospholipids is arachidonic acid (AA). AA represents another product of PLA_2_ and, consistently, its level also correlated with that of LPA (ρ = 0.58; Fig. 2B), while all other polyunsaturated fatty acids analyzed showed much weaker correlations (Fig. 2E).

These findings also suggest that multiple enzymes involved in the two‐step generation of LPA from phospholipids are coregulated in a subset of patients and determine the generation of LPA in the HGSC microenvironment. This conclusion is supported by the observation that the levels of autotaxin and its substrate LPC also showed a clear positive correlation (ρ = 0.62; *P* = 0.003; Fig. S4).

### 3.3 A major role for TAMs in the generation of extracellular LPA

**Figure 3:**
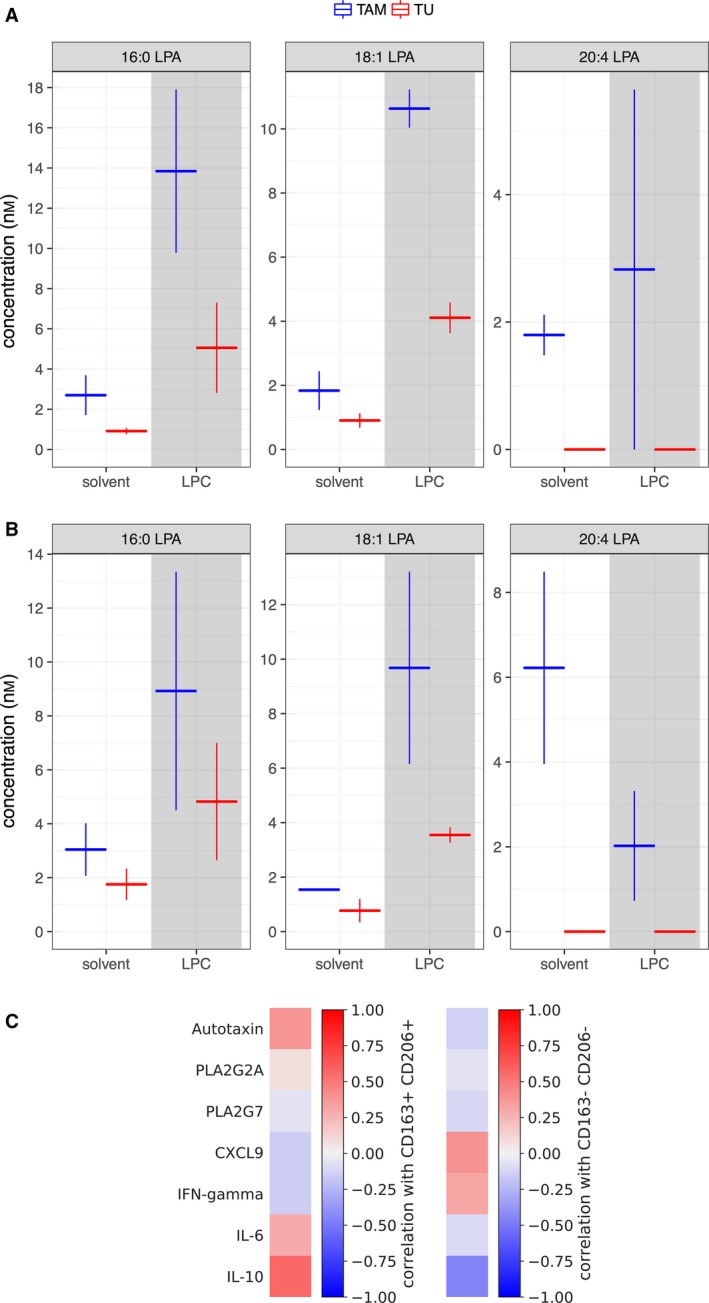
Role of TAMs and tumor cells in extracellular LPA production. (A, B) Levels of 16:0, 18:1, and 20:4 LPAs in culture supernatants from tumor cells or TAMs. Cells cultured in OCMI medium supplemented with 50% ascites for 24 h were incubated in ascite‐free medium containing 0.1% fatty acid‐free bovine serum albumin for an additional 24 h prior to adding either solvent or LPCs (16:0 and 18:1 mixture), and culture supernatants were analyzed by LC‐MS/MS 8 h (A) and 24 h (B) later. Horizontal bars represent the mean of two biological duplicates and vertical lines the range. (C) Heatmap depicting the correlation of the abundance of CD163+CD206+ (left) and CD163‐CD206‐ TAMs (right) in ascites with the ascites levels of the indicated LPA‐generating enzymes (Spearman correlation, ρ). IL‐6 and IL‐10 were included as known mediators prognostic of a poor survival in HGSC, and CXCL9 and IFNγ as cytokines prognostic of a favorable clinical outcome (Lieber *et al*., 2018; Reinartz *et al*., 2016; Worzfeld *et al*., 2017).

The major contribution of TAMs to the pool of autotaxin and PLA_2_ enzymes described above (Fig. 1B,C) point to a predominant role in the generation of extracellular LPA. To obtain direct experimental evidence for this hypothesis, we analyzed the production of LPC and LPA by ascites‐derived tumor cells and TAMs in serum‐free medium in the absence of exogenous lipids and in the presence of supplemented LPC (mixture of 16:0 and 18:1). As shown in Fig. 3A,B (left‐most bars), incubation of TAMs in lipid‐free medium resulted in a clear increase in the concentration of 20:4‐LPA at both 8 and 24 h, while no significant change was observed with tumor cells. Since no exogenous lipids were added, it is likely that TAMs use an endogenous pool of precursor molecules for extracellular 20:4‐LPA biosynthesis, consistent with the presence of lipid droplets in these cells (Schumann *et al*., 2015). The addition of 18:1‐LPC and 16:0‐LPC (shaded areas in all panels) leads to a clearly increased production of the respective LPA derivatives by both TAMs and tumor cells, with TAM remaining the main producers of these LPAs. These findings support a leading role for TAMs in LPA synthesis, and in particular of the 20:4 species. Our observations also suggest that LPC is rate‐limiting for the LPA production.

We next sought to identify potential links to TAM subpopulations with distinct functions and prognostic value. As previously reported, CD163 and CD206 are surface markers on TAMs in HGSC ascites associated with protumorigenic functions and a poor clinical outcome (Reinartz *et al*., 2014; Worzfeld *et al*., 2018). The analysis in Fig. 3C revealed a clear correlation of autotaxin levels in ascites and the abundance of CD163+CD206+ TAMs, while no correlation was observed for PLA_2_G2A or PLA_2_G7. This is consistent with the observation that autotaxin is mainly released by TAMs, while phospholipases are secreted by other cell types at substantial amounts as well (Fig. 1C). For comparison, we included in Fig. 3C mediators prognostic of a poor survival in HGSC (IL‐6, IL‐10) or linked to a favorable clinical outcome (CXCL9, IFNγ) (Lieber *et al*., 2018; Reinartz *et al*., 2016; Worzfeld *et al*., 2017).

### 3.4 Association of LPA with clinical outcome

**Figure 4:**
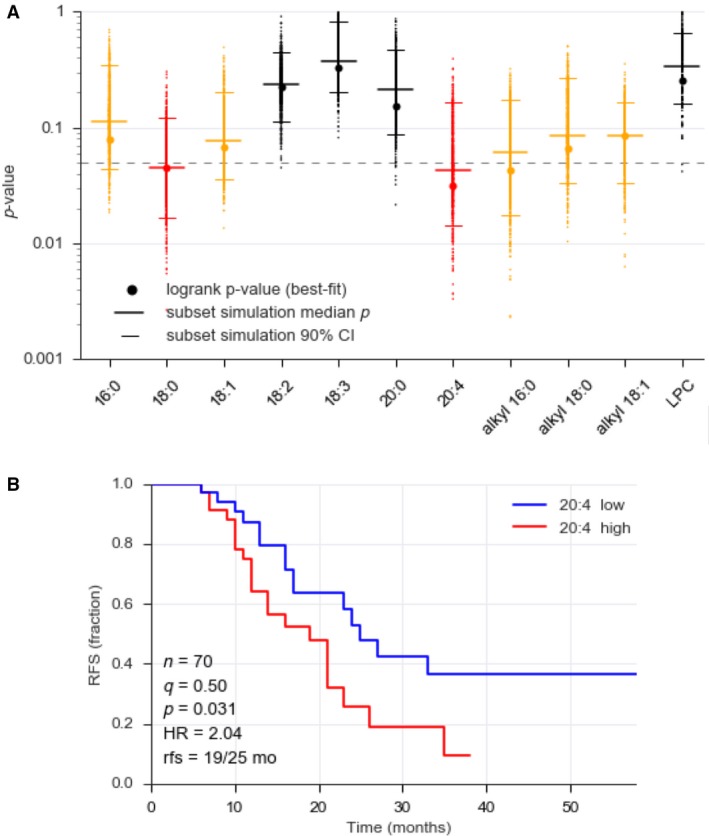
Association of LPA species in ascites with RFS. (A) Logrank test p‐values (best split) were determined for a cohort of 70 HGSC patients (filled circles). Dots (appearing as vertical lines at high densities) represent the results of subset simulation, where logrank *P*‐values were calculated for 1000 random drawings of samples representing 90% of the original dataset. Median *P*‐value for these simulations and 95% confidence intervals are shown as horizontal lines. Red: median *P*‐value < 0.05 and hazard ratio (HR) > 1, interpreted as a robust association with a short RFS; orange: median *P*‐value > 0.05, but lower CI limit < 0.05, and hazard ratio (HR) > 1, indicating a weak association with a short RFS; black: no significant association. (B) Kaplan–Meier plot exemplifying the association of 20:4 acyl‐LPA with RFS. q: quantile used for splitting datasets (high versus low); *P*: logrank test *P*‐value; rfs: median RFS times for high/low levels of 20:4 acyl‐LPA.

To address the clinical relevance of increased LPA levels in ascites, we determined potential links of the LPA species analyzed above with relapse‐free survival (RFS) of HGSC patients. As shown in Fig. S5, logrank test revealed a significant inverse association between LPA levels and RFS only for 18:0 acyl‐LPA, 20:4 acyl‐LPA, and 16:0 alkyl‐LPA (red bars). A common problem with small datasets is a potentially strong influence of few single samples on *P*‐values close to the significance threshold. We therefore tested the robustness of our results by an approach of subset simulation, where we calculated the logrank *P*‐values for 1000 drawings of datasets randomly generated by omitting 10% of the samples of the original dataset. A median *P*‐value < 0.05 was found for both 18:0 acyl‐LPA and 20:4 acyl‐LPA, indicating a robust statistical significance (Fig. 4A, red). 16:0 acyl‐LPA, 18:1 acyl‐LPA, and all three alkyl‐LPAs yielded slightly higher median *P* values, pointing to a weaker association with clinical outcome (Fig. 4A, orange). The other acyl‐LPAs (18:2, 18:3, 20:0) as well as LPC yielded no significant results. The inverse association of 20:4 acyl‐LPA is also illustrated by the Kaplan–Meier plot in Fig. 4B.

We have previously reported that the ascites level of AA is associated with a shorter RFS (Reinartz *et al*., 2016). Since AA and LPA are components of the same metabolic pathway, we assessed a potential prognostic interdependence of these lipid mediators by testing distribution probabilities. To this end, samples were split at their ‘best‐fit’ quantile (as in Fig. 4 and Fig. S5) and the significance of the overlaps was assessed by hypergeometric testing. This analysis yielded a *P*‐value of 0.00027, suggesting that AA and LPA are not independent prognostic factors of RFS. This is consistent with the correlation of AA and LPA levels in ascites (Fig. 2B). Taken together with our previously discovered association of PLA_2_G7 levels in ascites with a poor clinical outcome (Reinartz *et al*., 2016), our findings identify secretory PLA_2_ and its products as crucial determinants of HGSC survival. This is presumably due to the PLA_2_‐catalyzed generation of two (classes of) precursors of protumorigenic molecules, that is, lysophospholipids (as autotaxin substrates for LPA synthesis) and AA (eicosanoid metabolism or functions in its nonmetabolized form).

### 3.5 Promotion of three‐dimensional matrix invasion by all major LPA species in ascites

**Figure 5:**
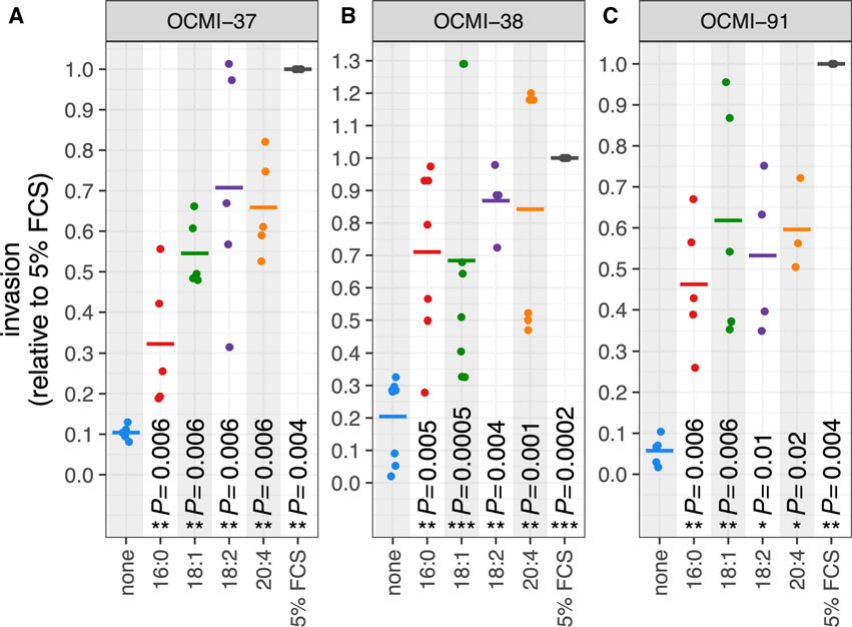
LPA‐induced Matrigel invasion by HGSC cells. Cancer cells derived from three different patients (panels A, B, and C) were analyzed for invasion into a three‐dimensional Matrigel matrix in response to distinct acyl‐LPA species used as attractants. Data represent the mean of 3–6 biological replicates (dots). Values were normalized to 1 for FBS‐induced invasion in each biological replicate. Horizontal bars show the mean. Asterisks indicate *P*‐values determined by one‐sided paired *t*‐test [LPA or FBS versus no attractant (none)]. *: *P* < 0.05, **: *P* < 0.01, ***: *P* < 0.001, ****: *P* < 0.0001.

The stimulation of cancer cell motility and invasion is presumably instrumental in LPA‐triggered HGSC progression (for reviews, see Jesionowska *et al*., 2015; Willier *et al*., 2013). We therefore sought to investigate the effect of distinct LPA species in a three‐dimensional matrix invasion assay. As shown in Fig. 5, HGSC cells efficiently invaded Matrigel matrices toward a gradient of FBS. A strong induction of invasion was also observed when defined acyl‐LPA species, that is, 16:0, 18:1, 18:2, or 20:4, were used as attractants (Fig. 5). The effect of different LPA species varied up to ~ 2‐fold for a given cell line, but there was no consistent pattern. We also performed analogous experiments addressing the role of LPA species in promoting HGSC motility (two‐dimensional migration), which yielded results consistent with those for Matrigel invasion (Fig. S6). Taken together, our findings support the conclusion that all major LPA species found in ascites play a role in promoting HGSC motility and invasion.

### 3.6 Cell type‐selective LPA receptor expression

**Figure 6:**
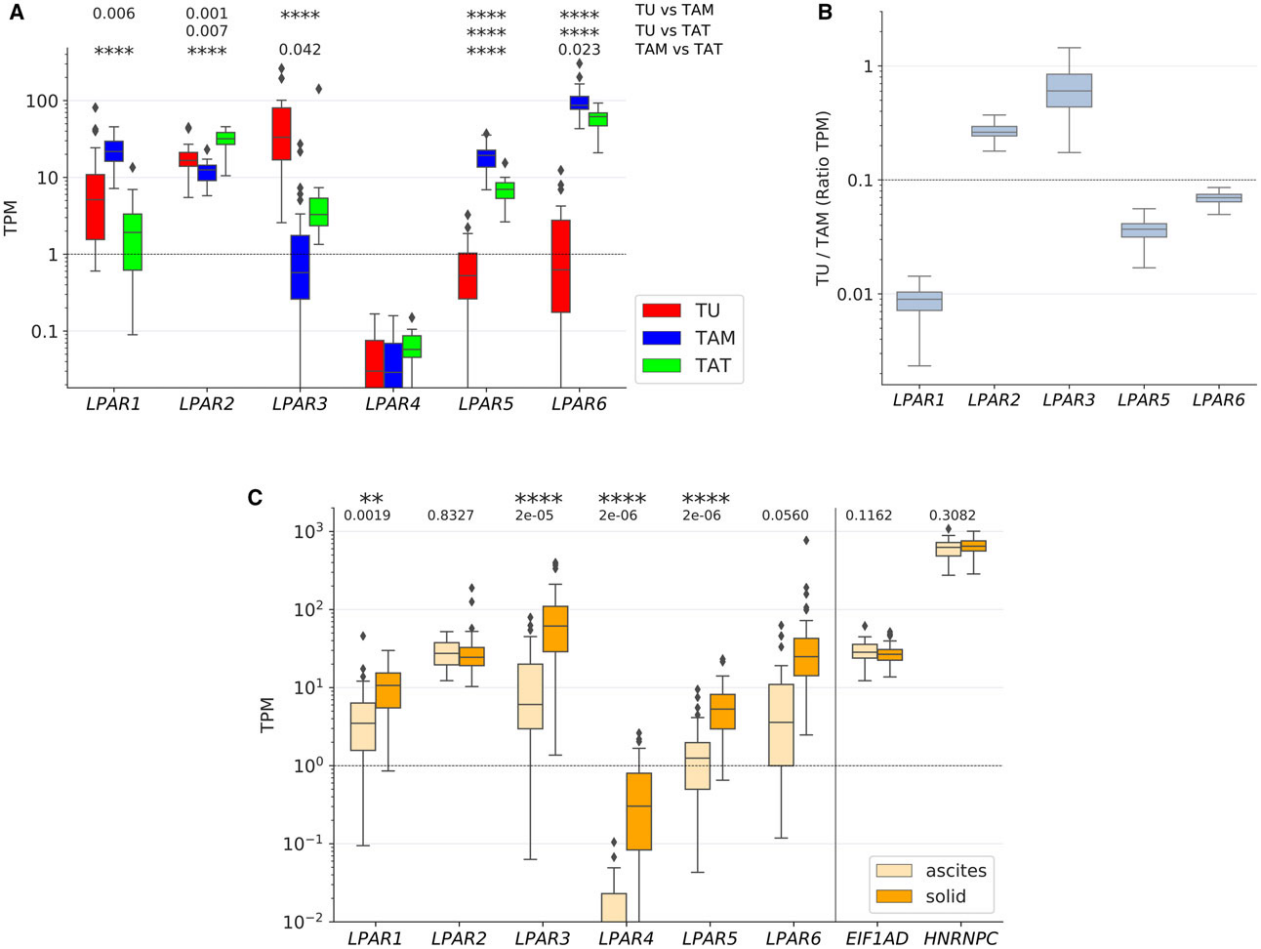
Differential expression and survival associations of LPA receptor (*LPAR*) genes. (A) Expression of genes coding for six members of the *LPAR* family in tumor cells (TU) (*n* = 23), TAMs (*n* = 32) and TATs (*n* = 8) from HGSC ascites (RNA‐Seq data). (B) Ratio of *LPAR* mRNAs in tumor cells versus TAMs (data from panel A) assessed by bootstrapping. (C) Evaluation of published RNA‐Seq data (Patch *et al*., 2015) analyzing the expression of *LPAR* expression in tumor cells from ascites (*n* = 29) and in solid tumor tissue (*n* = 82) from HGSC ascites. *EIF1AD* and *HNRNPC* (controls) were included as genes expressed at very similar levels in all samples irrespective of cell type and patient. Boxplots in (A) and (C) show medians (horizontal line in boxes), upper and lower quartiles (box), range (whiskers), and outliers (diamonds). *: *P* < 0.05, **: *P* < 0.01, ***: *P* < 0.001, ****: *P* < 0.0001 by unpaired *t*‐test.

We next sought to identify potential cell type‐selective LPA signaling mechanisms determined by the differential engagement of LPA receptor subtypes. Toward this goal, we analyzed the expression pattern of the six *LPAR* family members in tumor cells, TAMs and TATs. As shown in Fig. 6A, *LPAR4* is not expressed in any of these cell types at detectable levels. In tumor cells, *LPAR1, LPAR2,* and *LPAR3* are the major subtypes, while *LPAR5* and *LPAR6* are expressed only at very low levels in a subset of patients, if at all. In contrast, *LPAR5* and in particular *LPAR6* are major receptor subtypes in TAMs and TATs, besides *LPAR1* in TAMs, *LPAR2* in both TAMs and TATs, and *LPAR3* in TATs. This cell type‐specific pattern was confirmed by bootstrapping, as shown for TAMs in Fig. 6B. Taken together, these findings point to immune cell‐selective functions of LPAR5 and LPAR6, while tumor cells seem to engage primarily LPAR1‐3. A very similar expression pattern was found with published RNA‐Seq data (Patch *et al*., 2015) for solid tumor tissue and ascite‐derived cells from OC patients (Fig. 6C). The higher level of *LPAR5* and *LPAR6* in tumor tissue most likely reflects the presence of tumor‐associated immune cells, which is consistent with our own data. Taken together, our data strongly suggest that LPA triggers distinct signaling pathways in OC cells, TAMs and TATs.

### 3.7 Cell type‐selective transcriptional signaling induced by LPA

**Figure 7:**
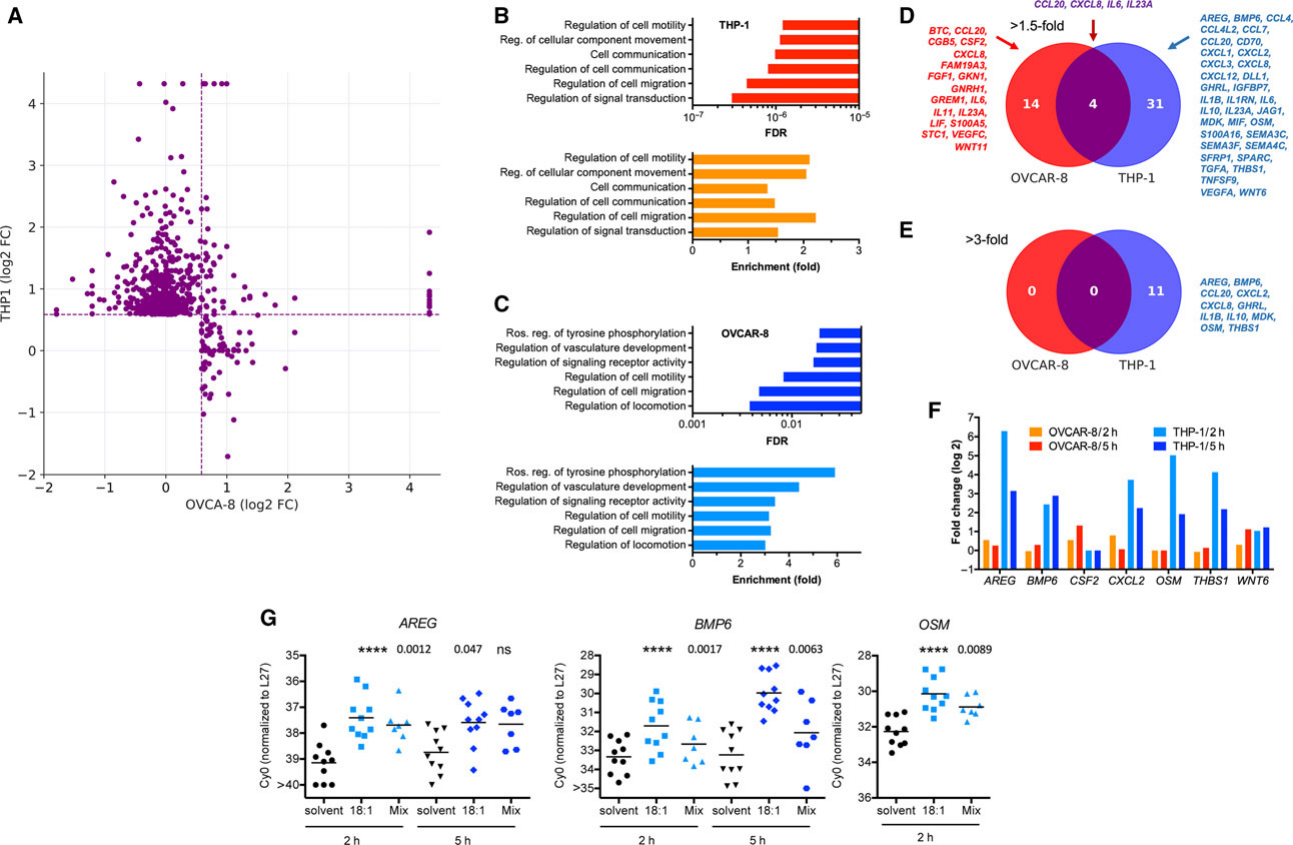
Cell type‐selective upregulation of genes by LPA in tumor cells and macrophages. (A) Expression of genes induced by LPA in THP‐1 macrophages and OVCAR‐8 cells (TPM > 1; FC > 1.5). inf: infinite due to lack of expression (TPM = 0) in either cell type. Cells were treated for 5 h with 5 μm LPA mix. (B, C) Gene ontology term enrichment analysis (http://geneontology.org) of LPA‐induced genes for THP‐1 cells (B) and OVCAR‐8 cells (C). FDR: false discovery rate; enrichment (fold): number of proteins induced by LPA versus random expectation. (D,   E) Genes coding for cytokines or growth factors by LPA in cells treated as in panel A (E: minimum FC = 1.5; F: minimum FC = 3). (F) Examples of cytokine genes induced by LPA in tumor cells or TAMs treated with LPA for 2 and 5 h (RNA‐Seq data). (G) Verification of RNA‐Seq data by RT‐qPCR for the indicated genes in THP‐1 cells treated with solvent, in 18:1‐LPA (5 μm) or an LPA mixture (Mix) containing the median concentrations of acyl‐LPAs in ascites (biological replicates: *n* = 10, except for OSM/Mix: *n* = 7. *P*‐values were determined by paired *t*‐test (****P < 0.0001).

To obtain further evidence for cell type‐selective mechanisms mediating LPA‐triggered signaling, we performed RNA‐Seq analyses of tumor cells and macrophages treated with an LPA mixture resembling the LPA composition in ascites. The HGSC cell line OVCAR‐8 (Hernandez *et al*., 2016; Schilder *et al*., 1990) and THP‐1 cells (Tsuchiya *et al*., 1980) differentiated to macrophages by PMA (Park *et al*., 2007) were used for this purpose. To minimize undesirable influences on measured gene expression patterns by PMA‐triggered signaling, cells were cultured for 5 days in normal medium after a short PMA treatment (4 h). As illustrated in Fig. 7A, 104 protein‐coding genes were induced in OVCAR‐8 cells (Table S3) and 713 genes in THP‐1 cells [TPM > 1; fold change (FC) > 1.5; Table S4] after a 5‐h treatment of serum‐deprived cells with a mixture of LPA species resembling the concentrations in ascites.

Gene ontology term enrichment analysis (http://geneontology.org) of these genes identified two major groups of significantly enriched terms, that is, (a) cell motility, migration, and movement and (b) cell communication/positive regulation of signal transduction and tyrosine phosphorylation (Fig. 7B,C). Consistent with this observation, we found 49 genes induced by LPA in OVCAR‐8 or THP‐1 cells (FC > 1.5) that encode growth factors or cytokines with only four genes upregulated in both cell types (Fig. 7D). Upon raising the threshold to FC = 3, the respective numbers were 11 for THP‐1 cells and 0 for OVCAR‐8 cells (Fig. 7E). Additional RNA‐Seq experiments showed that induction of the same genes was also seen (and frequently even higher) after 2‐h LPA treatment (Fig. 7F; Tables S3 and S4), suggesting that these genes are direct targets of LPA‐triggered signaling. Induction was generally much stronger in THP‐1 compared to OVCAR‐8 cells reaching values of 10‐ to 80‐fold for several genes (e.g., *AREG, BMP6, CXCL2, OSM, THBS1*). The RNA‐Seq data were verified by qRT‐PCR for *AREG, BMP6,* and *OSM* with using independent biological replicates (Fig. 7G).

Our data suggest that LPA‐induced genes in TAMs significantly contribute to the composition of the OC secretome. Consistent with this notion, most of the LPA‐induced cytokine and growth factor genes are expressed at substantial levels by OC TAMs (Worzfeld *et al*., 2018).

## 4 Discussion

In spite of its postulated role in OC progression, the origin of LPA within the tumor microenvironment and the clinical relevance of distinct LPA species remain obscure. In the present study, we show that distinct acyl‐LPA species are strongly elevated in the ascites from HGSC patients relative to plasma levels. While 16:0, 18:2, and 20:4 acyl‐LPAs are highly abundant, all alkyl‐LPAs analyzed were present at very low or even undetectable levels (Fig. 1A), suggesting that acyl‐LPAs may be clinically more relevant. This is consistent with our observation that the concentrations of both 18:0 and 20:4 acyl‐LPA in ascites are robustly associated with a short RFS (Fig. 4). Intriguingly, this clinical association is LPA species‐specific, since there is no detectable link of 18:2, 18:3, or 20:0 acyl‐LPA with the clinical outcome, suggesting that different acyl‐LPA species might differ in their impact on the signaling transduction network of their target cells. This notion would be compatible with the reported receptor selectivity of different LPA species (Tigyi, 2010). 16:0, 18:0, 18:1, and 18:2 acyl‐LPA have previously been reported to be elevated in OC ascites (Lu *et al*., 2002; Xiao *et al*., 2001), but the clinically highly relevant 20:4 species has not been described in ascites to date.

Our data indicate that all major LPA species found in ascites are able to promote HGSC motility and invasion *in vitro* to a similar extent (Fig. 5). Therefore, the differential effects of individual LPA species on RFS are presumably not related to their ability to promote cancer cell invasion. It is conceivable that other LPA‐regulated functions in cancer cells (see introduction) and/or a possible impact of LPA on tumor‐associated host cells play a role in this context. As suggested by our RNA‐Seq analysis (Fig. 7) and a previous publication describing an effect of LPA on monocytic differentiation (Ray and Rai, 2017), macrophages represent a candidate cell type for mediating this effect.

Our transcriptome and secretome analyses of primary ascite‐derived tumor cells, TAMs and TATs provided insight into the pathways involved in LPA synthesis and the role of different cell types. Thus, the major route of LPA synthesis appears to be the consecutive action of secretory phospholipases A_2_ (PLA_2_), subtypes G2A, G7, and G12A, followed by the phospholipase D‐like autotaxin, since the gene coding for LIPI (as the essential enzyme of the alternative pathway; Fig. 2A) is not expressed in the three major cell types in ascites (Fig. 1B). Our data also show that the enzymes of the PLA_2_–autotaxin pathway as well as the intermediate metabolite LPC are coordinately upregulated in ascites (Fig. 2). It appears that TAMs play an essential role in this pathway, since they are the main producers of PLA_2_G7 and autotaxin (Fig. 1B). This conclusion is substantiated by MS‐based metabolomic analyses, which showed that primary TAMs, but not tumor cells, produce 20:4 acyl‐LPA in short‐term cultures (Fig. 3A,B). We have previously reported that CD163+CD206+‐positive TAMs are associated with protumorigenic features and a shorter RFS in HGSC (Adhikary *et al*., 2017; Reinartz *et al*., 2014; Worzfeld *et al*., 2018). Intriguingly, correlation analyses revealed these macrophages as the main producers of autotaxin and PLA_2_G2A (Fig. 3A), providing another possible explanation for the protumorigenic properties of CD163+CD206+‐positive TAMs.

We also analyzed potential correlations of LPA levels with tumor or TAM content in ascites. The analyses showing that neither the number of tumor cell spheroids of tumor cells nor the abundance of TAMs (CD14^+^ cells of total leukocyte count) in ascites was correlated with the concentration of any of the LPA species (data not shown). This suggests that LPA levels are determined by the entire tumor microenvironment, including solid tumor tissue, rather than merely by ascites cells.

Our transcriptomic data revealed further evidence for cell type selectivity with respect to LPA signaling. While primary tumor cells express predominantly genes encoding EDG‐type receptors (LPA1‐3), immune cells (TAMs and TATs) also express genes for the non‐EDG receptors LPAR5 and LPAR6 at high levels (Fig. 6). These receptors drive common as well as subtype‐specific signaling transduction pathways (Yung *et al*., 2014). Consistent with these differential effects on intracellular signaling and the observed cell type selectivity of LPAR subtype expression, RNA profiling of LPA‐stimulated OVCAR‐8 cells and THP‐1 macrophages revealed a very small overlap of target genes (Fig. 7C–E). Functional annotation of these genes identified cell motility and migration as well as cell communication and signaling receptor activity as the most significantly associated terms, suggesting that LPA contributes to cancer spread via the tumor secretome. These findings are consistent with previous publications, which identified *IL6* (Fang *et al*., 2004), *CXCL1* (Lee *et al*., 2006) and *CXCL8* (Fang *et al*., 2004; Schwartz *et al*., 2001) as LPA target genes in cancer cells.

## 5 Conclusions

Our data show that LPA in the ovarian cancer microenvironment is produced via phospholipase PLA_2_ and autotaxin secreted by TAMs. LPA induces the production of growth factors and cytokines and triggers matrix invasion, associated with an early disease recurrence in patients. The 20:4 LPA species appears to play a major role in this context. Our findings point to several potential strategies to interfere with LPA‐triggered signaling and its impact on cancer progression. Besides the previously proposed inhibition of PLA_2_ and autotaxin, these include the blockade of TAM functions and the interference with specific LPA receptor‐driven signaling pathways. To be able to evaluate these options, it will be necessary to elucidate the underlying molecular pathways and mechanisms in detail.

## Conflict of interest

The authors declare no conflict of interest.

## Author contributions

SR, TW, SM‐B, and RM designed the study, oversaw the project and wrote the paper. SR carried out cell culture experiments for secretome and lipid analysis and derived tumor cells from patients. SL performed experiments for RNA‐Seq analyses and qRT‐PCR with tumor cells and macrophages. AK and JG performed proteomic analyses and evaluated the data; DB and RG performed and evaluated Matrigel invasion assays; AA performed and evaluated migration and viability assays; JP, BW, and WAN carried out LC‐MS/MS analyses for LPA and LPC and evaluated the data; AN and TS established NGS methodologies and acquired the RNA‐Seq data; JMJ and UW provided clinical samples and analyzed clinical data. FF and RM performed bioinformatic and biostatistical analyses. All authors reviewed the results and approved the final version of the manuscript.

## Supporting information


**Fig. S1**. Levels of LPA species in plasma samples from HGSOC patients and patients with non‐malignant disease.
**Fig. S2**. Ratio of LPA levels in HGSC ascites versus plasma.
**Fig. S3.** Spearman correlation of the levels of LPA species in ascites.
**Fig. S4.** Correlation of LPC and autotaxin levels in ascites.
**Fig. S5**. Association of LPA species and LPC with RFS.
**Fig. S6**. Effect of LPA species on OC migration in a two‐dimensional transwell assay.

**Table S1.** Clinical data of HGSC patients included in the present study.
**Table S2.** MRM mass transitions and retention times for LPA and LPC analytes and internal standards.
**Table S3.** Genes upregulated by LPA in OVCAR‐8 cells.
**Table S4.** Genes upregulated by LPA in differentiated THP‐1 cells.
