# Supplementary material for: Cell type‐selective pathways and clinical associations of lysophosphatidic acid biosynthesis and signaling in the ovarian cancer microenvironment
Source: Mol Oncol. 2018 Nov 15;13(2):185–201. doi: 10.1002/1878-0261.12396 (PMC6360368; doi:10.1002/1878-0261.12396)
Supplement: Supplementary file 1 — Fig. S1. Levels of LPA species in plasma samples from HGSOC patients and patients with non‐malignant disease. Fig. S2. Ratio of LPA levels in HGSC ascites versus plasma. Fig. S3. Spearman correlation of the levels of LPA species in ascites. Fig. S4. Correlation of LPC and autotaxin levels in ascites. Fig. S5. Association of LPA species and LPC with RFS. Fig. S6. Effect of LPA species on OC migration in a two‐dimensional transwell assay. [file MOL2-13-185-s001.pdf]

Supporting Figures

Reinartz et al.: Cell-type-selective pathways and clinical associations of lysophosphatidic acid biosynthesis and signaling in the ovarian cancer microenvironment

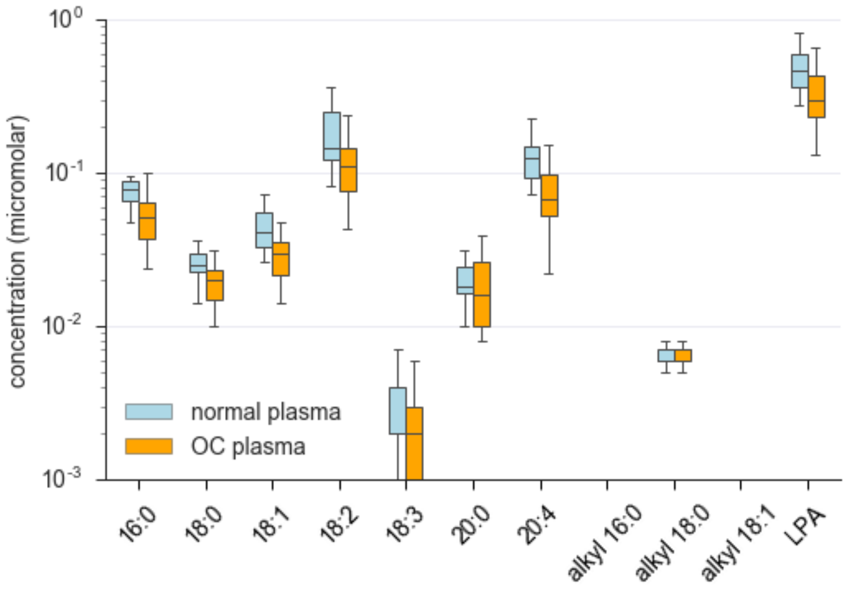

Figure S1.

Levels of LPA species in plasma samples from HGSC patients (n=19) and patients with non-malignant disease (ovarian cysts or myomatosis uteri; n=11). **: p<0.01, ****: p<0.0001 by paired t-test.

Supporting Figures

Reinartz et al.: Cell-type-selective pathways and clinical associations of lysophosphatidic acid biosynthesis and signaling in the ovarian cancer microenvironment

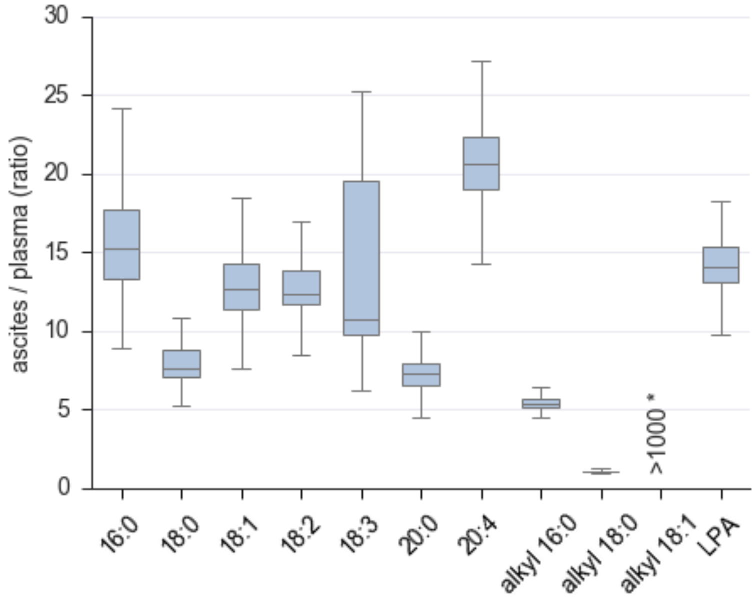

Figure S2.

Ratio of LPA levels in HGSC ascites (n=91) versus plasma (n=30). The graph shows the results of a bootstrapping analysis (1000 simulations): median of bootstrapping results (horizontal lines within boxes), upper and lower quartiles (box) and range (whiskers).

Supporting Figures

Reinartz et al.: Cell-type-selective pathways and clinical associations of lysophosphatidic acid biosynthesis and signaling in the ovarian cancer microenvironment

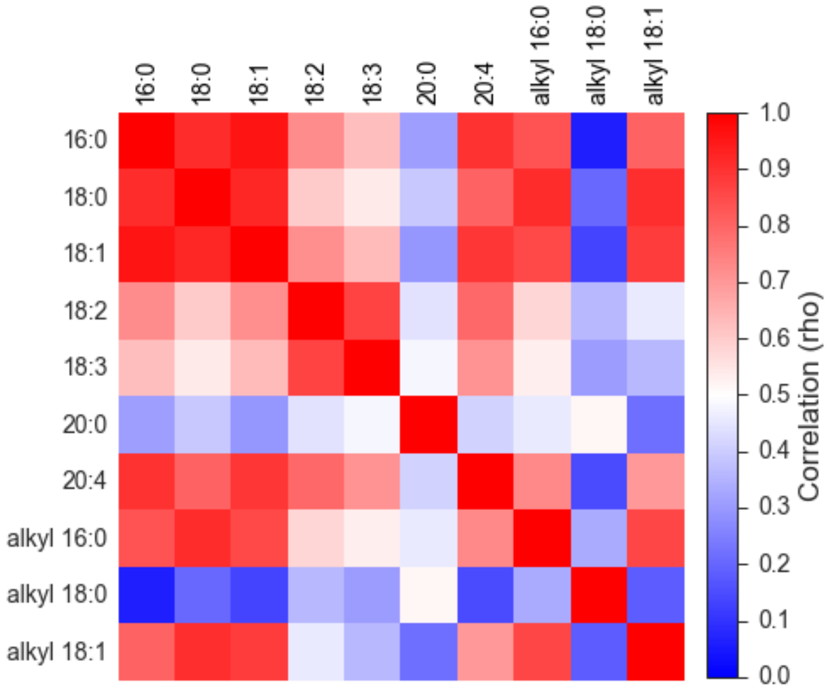

Figure S3.

Spearman correlation of the levels of acyl-LPA (16:0 … 20:4) and alkyl-LPA species in ascites.

Supporting Figures

Reinartz et al.: Cell-type-selective pathways and clinical associations of lysophosphatidic acid biosynthesis and signaling in the ovarian cancer microenvironment

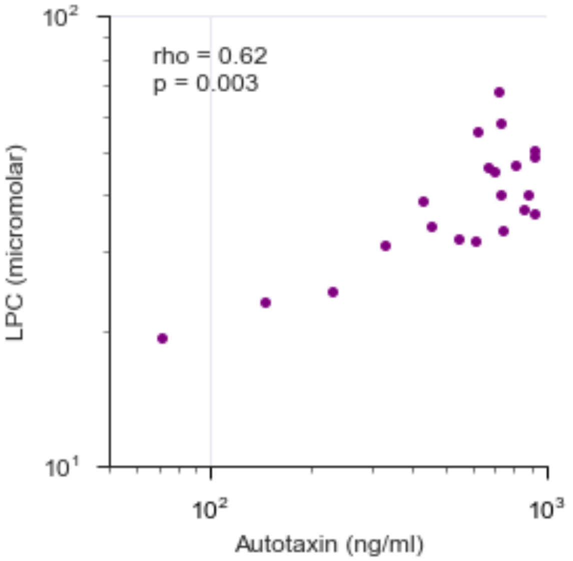

Figure S4.

Correlation of LPC and autotaxin levels in ascites. Rho: Spearman correlation coefficient.

Supporting Figures

Reinartz et al.: Cell-type-selective pathways and clinical associations of lysophosphatidic acid biosynthesis and signaling in the ovarian cancer microenvironment

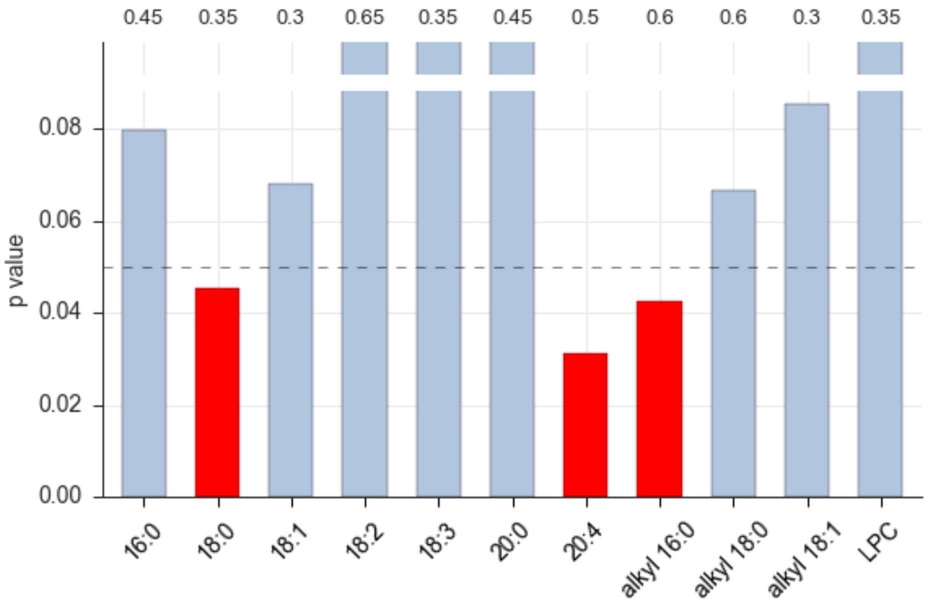

Figure S5.

Association of the levels of individual LPA species and LPC with the RFS of HGSC patients. Bars shows logrank test p values for splitting the data at the best fitting quantile (as indicated at the top). Red: significant inverse association (positive hazard ratio); light blue: nor significant association.

Supporting Figures

Reinartz et al.: Cell-type-selective pathways and clinical associations of lysophosphatidic acid biosynthesis and signaling in the ovarian cancer microenvironment

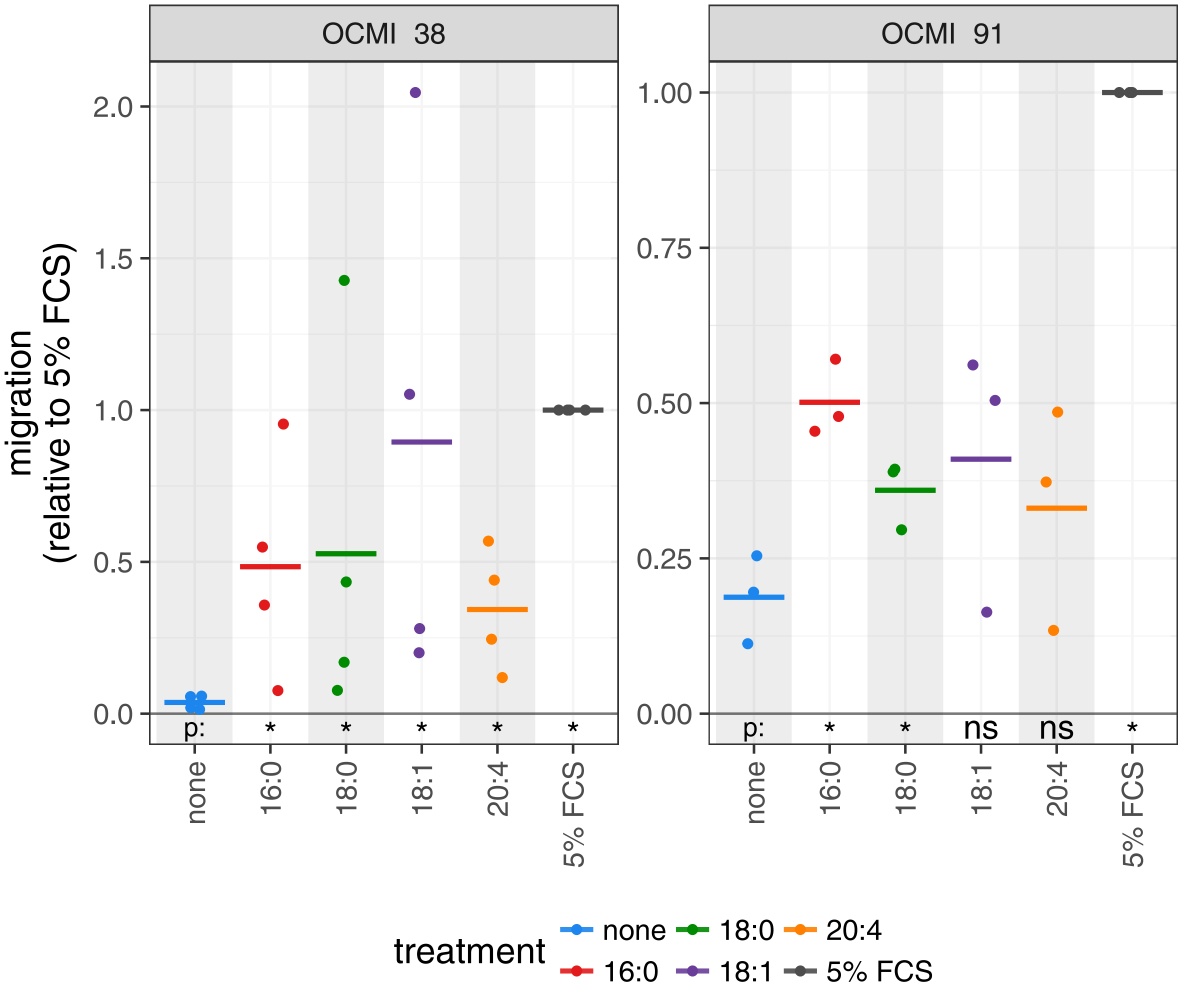

Figure S6.

Effect of LPA species on OC migration in a two-dimensional transwell assay as described in Experimental Procedures. Experimental details were as in Figure 5.
